# Kirschner wire reconstruction of medial and lateral column periosteal hinge in the treatment of multidirectionally unstable supracondylar fracture of the humerus in children

**DOI:** 10.1186/s40001-023-01560-2

**Published:** 2023-12-12

**Authors:** Hai-Long Ma, Xi-Wei Sun, Fang Liu, Zhong Tuo Hua, Jun Sun, Si-Cheng Zhang

**Affiliations:** Department of Pediatric Orthopedics, Anhui Provincial Children’s Hospital (Children’s Hospital of Anhui Medical University), No. 39 Wangjiang East Road, Hefei, 230051 Anhui People’s Republic of China

**Keywords:** Child, Supracondylar humeral fractures, Closed reduction, Percutaneous fixation

## Abstract

**Aim and objective:**

To compare the clinical effect of reconstruction of internal and lateral column periosteal hinge-assisted treatment with Kirschner wire and internal fixation with Kirschner wire in the treatment of multidirectional unstable supracondylar fractures of humerus in children.

**Methods:**

A retrospective cohort study was conducted to analyze the clinical data of 48 patients (31 male, 17 female; mean age: 6.7 ± 2.4 years old) with multidirectionally unstable supracondylar fractures of the humerus treated in our Hospital from August 2020 to August 2022. Twenty-five cases were treated with Kirschner wire reconstruction of the internal and lateral column periosteal hinge assisted by closed reduction and Kirschner wire internal fixation (study group). Twenty-three cases were treated with closed reduction and Kirschner wire internal fixation (control group). The operation time, intraoperative fluoroscopy times, percentage of patients who underwent open reduction after failure of closed reduction, fracture healing time, Baumann angle (BA), shaft-condylar angle (SCA), range of motion (ROM), and Flynn score of elbow at the last follow-up were compared between two groups. Complications such as infection and irritation of Kirschner wire tail were observed in two groups 2 months after the operation.

**Results:**

All patients were followed up for 10–22 months ([13.85 ± 2.89] months). The average operation time of the control group was 82.1 min, which was significantly longer than that of the study group 32.3 min (*P* < 0.05). The number of intraoperative fluoroscopy (29.4 ± 9.2) in the control group was significantly higher than that in the study group (15.2 ± 6.3) (*P* < 0.05). The incision rate of the control group was 17% while that of the study group was 0 (*P* < 0.05). According to Flynn score, the excellent and good rate of the elbow joint in the control group was 86.9% (20/23). The excellent and good rate of the elbow joint in the study group was 92.0% (23/25) (*P* > 0.05). There was no significant difference in fracture healing time, BA, SCA, and ROM between the two groups (*P* > 0.05). No infection or Kirschner wire tail irritation occurred in the two groups during the 2-month follow-up.

**Conclusion:**

Reconstruction of internal and lateral periosteal hinges with Kirscher wire has similar effects to closed reduction and Kirschner wire fixation in the treatment of multidirectionally unstable supracondylar fractures of the humerus in children, but it can shorten the operation time and reduce intraoperative fluoroscopy times and incision rate.

## Introduction

The most common elbow fracture in children is supracondylar fracture of the humerus, and the extended fracture is the most common [1–4]. Currently, the Gartland classification is most widely used in clinical practice [5]. For Gartland types I and IIA, outpatient manual reduction and plaster external fixation are used. Gartland type IIB and type III fractures are mainly treated by closed reduction and percutaneous pinning. In 2006, Leitch et al. [6] reported a special type of fracture. The complete fracture of the periosteum hinges at the broken end, lacking support at the broken end, and showing instability in the extension of the elbow joint. This type accounted for about 3% of all supracondylar fractures of the humerus in children with displaced fractures and was named the modified Gartland type IV fracture, also known as “multidirectionally unstable supracondylar fractures of the humerus.” There is a lot of overlap between Gartland type IV and flexion type supracondylar fractures of the humerus in children [7, 8]. Most supracondylar fractures of the humerus in children with flexion type can also be considered as Gartland type IV. The treatment of such fractures is one of the most challenging tasks for orthopedic surgeons. The goal of treating supracondylar fractures of the humerus in children is to achieve anatomical reduction and reduce the incidence of complications as much as possible. In view of the particularity of this kind of fracture, it is impossible to obtain stable fracture reduction before intraoperative Kirschner wire placement. Conventional closed reduction methods usually fail to maintain stability after reduction of such fractures and may even require repeated intraoperative reduction, thereby making it difficult to achieve satisfactory results. Eventually, open reduction has to be performed. In recent years, new surgical methods have been used to improve the success rate of minimally invasive treatments for this fracture type, but there is still no unified surgical method available. Therefore, we tried a variety of reduction methods during the operation. We found that the fracture was stabilized by using Kirschner wires inserted into the proximal humeral medullary space from both the medial and lateral sides of the distal fracture. We can consider the distal and proximal fracture and Kirschner wires as a whole. By using this technique, the distal and proximal ends of the fracture can be well reduced by relying on the Kirschner wire as the periosteal hinge.

## Clinical data and methods

### General information

The inclusion criteria were as follows: (1) multidirectionally unstable supracondylar fractures of the humerus with definite preoperative or intraoperative fluoroscopy imaging diagnosis; (2) ≤ 14 years old; (3) no manual reduction and nonsurgical treatment were performed before surgery; (4) not accompanied by other injuries or underlying diseases; (5) postoperative follow-up time, ≥ 6 months; and (6) availability of complete medical records. The exclusion criteria were as follows: (1) open fracture; (2) pathological fracture; (3) complicated with vascular or nerve injury or osteofascial compartment syndrome; and (4) multiple fractures, craniocerebral injury, or chronic disease.

A total of 48 patients (31 male and 17 female; mean age: 3–13 [6.90 ± 2.43] years old) with multidirectionally unstable supracondylar fractures of the humerus were included. They were divided into two groups according to the development of surgical techniques in our hospital. From August 2021 to August 2022, 25 cases were treated with Kirschner wire reconstruction of the internal and lateral column periosteal hinge assisted by closed reduction and Kirschner wire internal fixation (study group), and 23 cases were treated with closed reduction and Kirschner wire internal fixation (control group).

### Surgical methods

All operations were performed by the same group of surgeons. The patient was placed in the supine position, and after general anesthesia, the receiver of the mobile C-arm X-ray machine (Siemens Medical Instrument Co., Ltd., Germany) was close to the edge of the operating bed, and the C-arm X-ray machine receiver was used to replace the operating table. Lead clothing was applied to the neck, chest, abdomen, and pelvis of the patient, and semi-aseptic technology was used for routine disinfection [9]. The surgeon and an assistant simultaneously apply traction to the fracture site from both ends. If the skin in front of the elbow is depressed and shows ecchymosis or the fracture ends are stuck, the “milking” method can be tried to restore the stuck fracture ends [10].

**Fig. 1:**
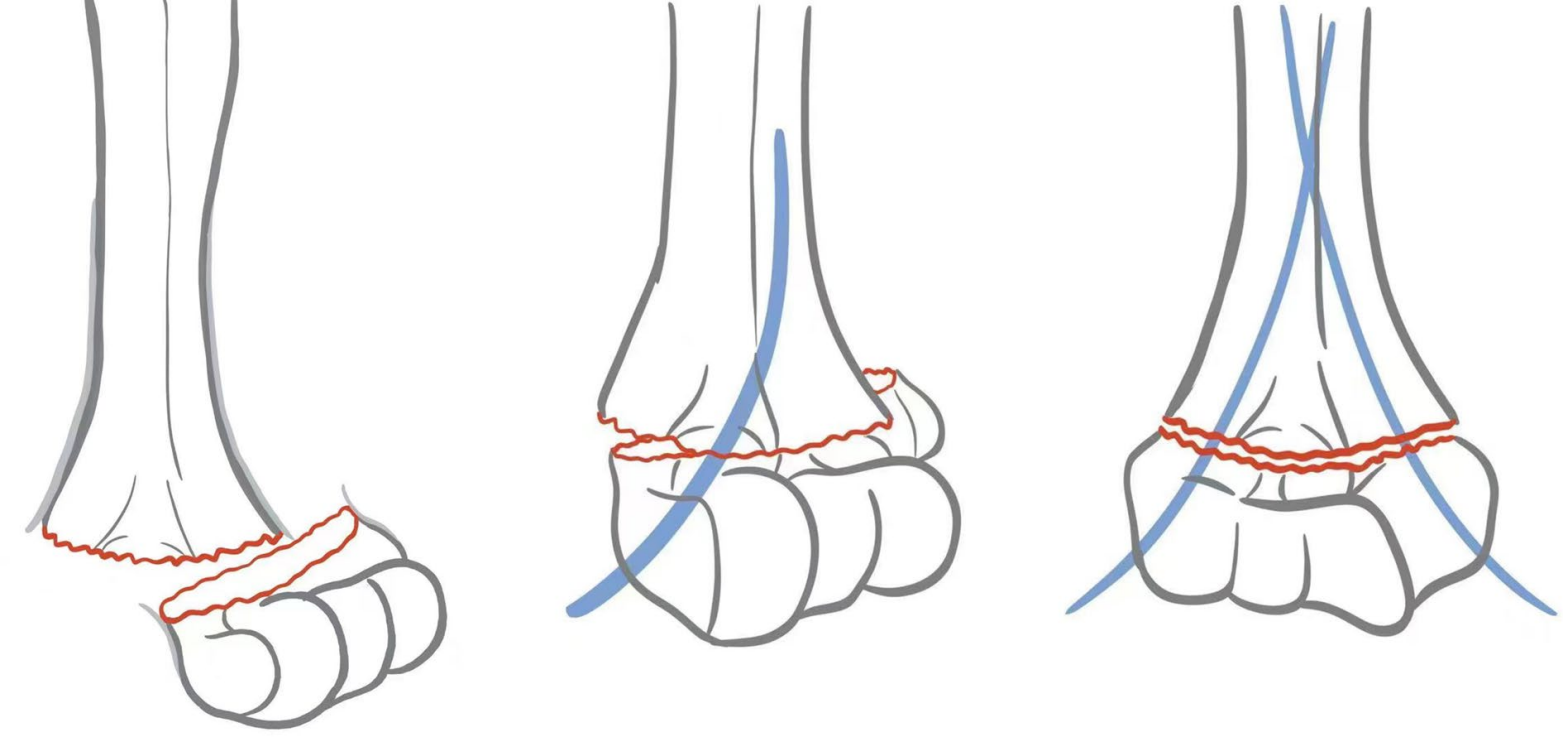
Schematic diagram of Kirschner wire reconstruction of medial and lateral column periosteal hinge

Study group: With continued traction, the X-ray confirmed that the fracture was unstuck. The distal end of the fracture was showed extreme ulnar skew, and a 2.0-mm-diameter Kirschner wire was drilled into the proximal medullary cavity with an electric drill from the lateral condyle of the distal humerus (the above procedure can also be performed by the surgeon alone). The C-arm X-ray machine confirmed that the Kirschner wire had entered the proximal medullary cavity, and then, the distal end of the fracture was extremely radially skewed. A 1.5-mm or 2.0-mm Kirschner wire was drilled using an electric drill from the distal medial epicondyle of the humerus (the ulnar nerve should be protected) into the proximal medullary cavity of the fracture. A 2.0-mm Kirschner wire was selected for children ≥ 6 years old, and 1.5-mm Kirschner wire was selected for children < 6 years old. At this point, the distal, broken, and proximal ends of the fracture are viewed as a whole with the Kirschner wire as a bridge, so that the Kirschner wire acts as a temporary periosteal hinge to reconstruct the periosteal hinge of the distal internal and lateral pillars of the humerus. The Kirschner wire stabilizes the fracture end and can correct displacement. After cross placement, flexion, and extension of the elbow joint were performed, and fluoroscopy of the C-arm X-ray machine was used to verify the stability of the fracture. The Kirschner wire in the medullary cavity could be either removed or retained. The operation process is shown in Fig. 1.

Control group: Due to the complete instability of the broken end of the fracture, the assistant applied external force from the radio-lateral ulnar side (radio-lateral humerus supracondylar fracture) or ulnar side (ulnar lateral humerus supracondylar fracture) to correct the coronal displacement and maintain the reduction. The surgeon first placed two Kirschner wires from the lateral condyle of the humerus. Anteroposterior and lateral elbow fluoroscopy was performed under the C-arm. If the fracture is well reduced, continue to insert Kirschner wire on the ulnar side. If the reduction of the fracture is unsatisfactory, open reduction should be used instead.

### Postoperative management

All patients underwent plaster fixation after surgery. The degree of swelling, finger sensation, and activity of the affected limb were observed during hospitalization. Regular postoperative dressing changes were carried out, and patients were discharged when no obvious exudation and swelling were observed. After the discharge, the dressing was changed regularly in the outpatient department and the Kirschner wire tail exudation was observed. Four weeks after the surgery, an outpatient review was carried out. At this time, if the positive and lateral radiographs showed rich callus growth, the plaster could be removed, the Kirschner wire pulled out, and the patient advised to follow active functional exercise.

### Observation indices

The operation time, intraoperative fluoroscopy times, proportion of patients who underwent open reduction due to closed reduction failure (hereinafter referred to as the incision rate), fracture healing time, Baumann angle (BA), shaft-condylar angle(SCA), range of motion(ROM), and Flynn score of elbow at the last follow-up were compared between the two groups [11]. Complications such as infection and skin irritation of Kirschner wire tail were supervised in the two groups 2 months after operation.

### Statistical analysis

SPSS25.0 statistical software (IBM Corporation, Armonk, NY, USA) was used for all statistical analysis. Kolmogorov–Smirnov method was used to test for data normality. Measurement data conforming to the normal distribution were expressed as $\overline{x}$ ± *s*. For comparison between groups, the *t*-test of two independent samples was used; for measurement data not conforming to the normal distribution, M (Q1, Q3) was calculated to reflect the difference between the two groups and Mann–Whitney U test was used for comparison between groups. Statistical data were expressed as a percentage, and chi-square test was used for comparison between groups. All tests were two-sided, and *P* < 0.05 was considered to indicate statistically significant differences.

## Results

**Fig. 2:**
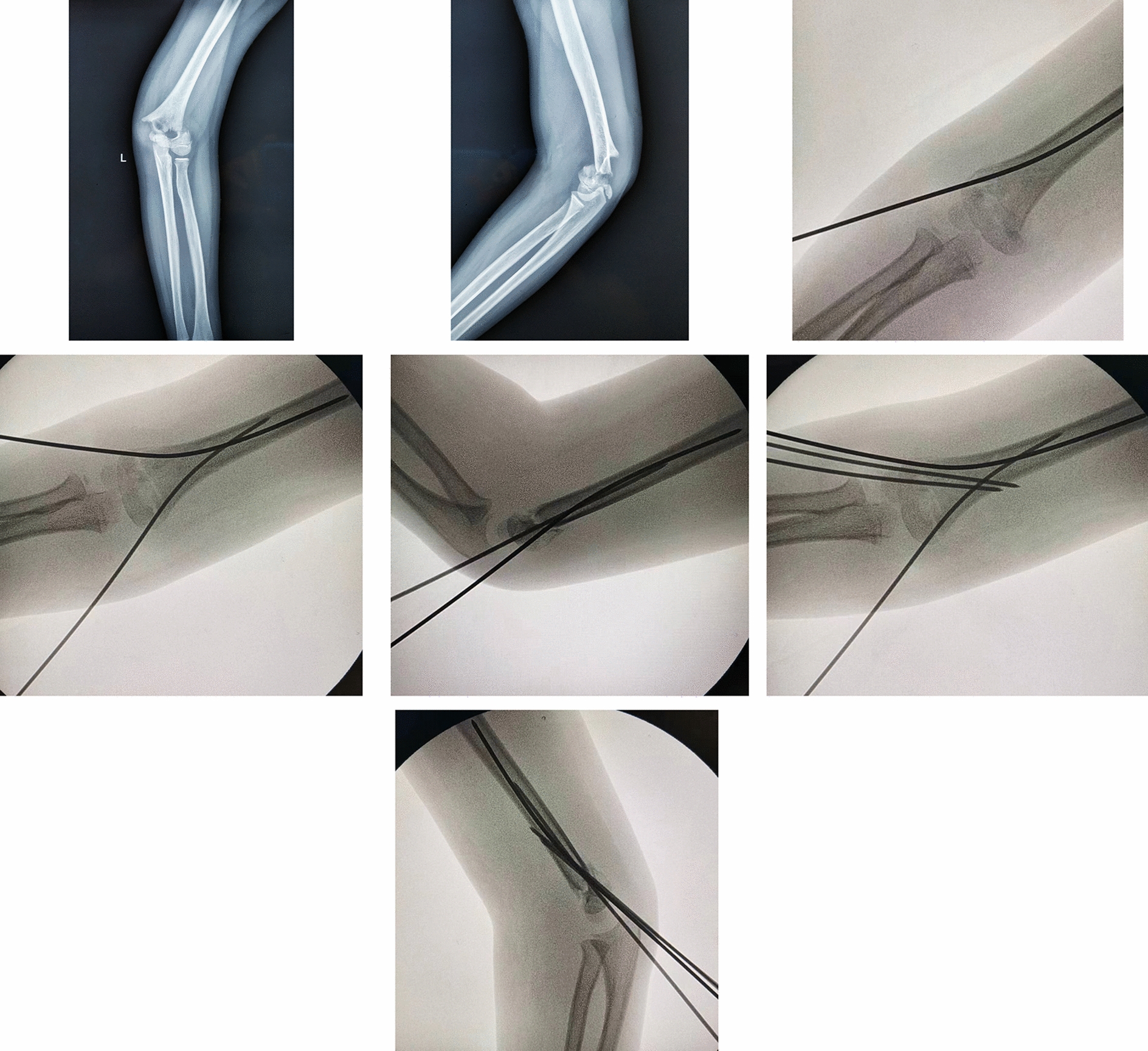
Preoperative, intraoperative, and postoperative fluoroscopic images

The comparison of general data between the two groups showed no statistical significance (all *P* > 0.05), indicating comparability (Table 1). All fractures in the study group were successfully closed reduction, and four cases in the control group were changed to open reduction. The difference between the two groups was statistically significant (*P* < 0.05). The operation time and intraoperative fluoroscopy times of the study group were less than those of the control group, and the differences were statistically significant (*P* < 0.05) (Table 2). Patients in both groups were followed up. The study group was followed up for 10 to 22 months, with an average of 13.8 ± 2.8 months. The control group lasted from 10 to 20 months, with an average of 13.9 ± 3.0 months. Imaging review showed that the fractures of both groups healed, and there was no significant difference in healing time, BA, SCA, and ROM (*P* > 0.05). 0.3 months after operation, five children were limited in the flexion and extension of the elbow joint, they were instructed to take active functional exercise, and all returned to the normal range of motion at the last follow-up. According to Flynn’s score at the last follow-up, the excellent and good rate of elbow joint function was 92.0% (23/25) in the study group and 86.9% (20/23) in the control group, with no statistical significance (*P* > 0.05) (Table 3). None of the patients saw the doctor again due to complications such as infection and irritation of the tail of Kirschner needle. Typical cases are shown in Fig. 2.

**Table 1:** Comparison of general data of patients with multidirectionally unstable supracondylar fracture of the humerus between the two groups

Group	Total (*n*)	Sex		Age (years, $\overline{x}$ ± *s*)	Profile		Time from injury to operation (h, $\overline{x}$ ± *s*)
		Male (*n*)	Female (*n*)		Left (*n*)	Right (*n*)	
Study group	25	18	7	7.12 ± 2.24	13	12	39.08 ± 29.85
Control group	23	13	10	6.65 ± 2.64	13	10	35.87 ± 16.24
Statistic value	–	1.255		0.664	0.099		0.457
P-value	–	0.263		0.510	0.753		0.650

**Table 2:** Comparison of operative time, intraoperative fluoroscopy times, fracture healing time, and open reduction in patients with multidirectionally unstable supracondylar fractures of the humerus between the two groups

Outcome indicator	Study group (*n* = 25)	Control group (*n* = 23)	Statistic value	*P*-value
Operation time ($\overline{x}$ ± s, mins)	32.32 ± 10.25	82.13 ± 50.44	− 4.648	< 0.001
Intraoperative fluoroscopy times ($\overline{x}$ ± s, times)	15.24 ± 6.25	29.35 ± 9.20	− 6.257	< 0.001
Open reduction (Y/N)	25/0	19/4	–	0.046

**Table 3:** Comparison of postoperative follow-up

Variables	Study group (*n* = 25)	Control group (*n* = 23)	Statistic value	*P*-value
Flynn score (excellent/good/fair/poor)	23/2/0/0	20/3/0/0	− 0.565	0.572
Baumann’s angle ($\overline{x}$ ± *s*, degrees)	73.10 ± 2.28	73.23 ± 2.65	− 0.177	0.160
Shaft-condylar angle ($\overline{x}$ ± s, degrees)	36.84 ± 3.81	38.23 ± 4.05	− 1.138	0.261
Range of motion ($\overline{x}$ ± s, degrees)	136.56 ± 6.49	137.61 ± 5.11	− 0.618	0.539
Follow-up time ($\overline{x}$ ± s, months)	13.84 ± 2.81	13.87 ± 3.04	− 0.035	0.972
Fracture healing time ($\overline{x}$ ± s, weeks)	4.28 ± 0.61	4.61 ± 1.12	− 1.248	0.221

## Discussion

The treatment of multidirectionally unstable supracondylar fractures of the humerus in children is challenging. Due to complete fracture of periosteal hinge, unstable fracture end, prolonged operation time, increased fluoroscopy times, high incision rate, and difficult closed reduction, it is challenging to achieve ideal reduction requirements for conventional closed reduction and Kirschner wire puncture treatment for such fractures [12–14]. To improve the treatment effect of this type of fracture, researchers and clinicians worldwide continue to explore surgical methods such as common lever-assisted reduction technology, prying reduction technology, and joystick reduction technology, but no consensus has yet been reached [15, 16]. The results of this study showed that the reconstruction of internal and lateral column periosteal hinges with Kirschner wire-assisted closed reduction and internal fixation with Kirschner wire could effectively reduce the operation time, reduce the number of fluoroscopies, reduce the incision rate, and improve the success rate of minimally invasive surgery.

The results of our study showed that both groups of patients achieved satisfactory efficacy. The main advantage of this method is the shortened operation time. This method increased the stability of the broken end of the fracture. After traction, the Kirschner wire was inserted both into the distal lateral condyle and the medial epicondyle of the fracture, through the fracture end, and into the pulp cavity of the proximal end of the fracture. The distal and proximal ends of the fracture were fixed as a whole with Kirschner wire as the support. This procedure resulted in improved coronal reduction of the fracture. Silva et al. [10] found that multidirectional unstable supracondylar humerus fractures (type IV) in children required longer operation time, averaging 70 min (range 49–96 min). In this study, the average operation time of the control group was 82.1 min, while that of the study group was 32.3 min, which was significantly less than that of Silva et al. In conclusion, Kirschner wire reconstruction of internal and lateral column periosteal hinges for the treatment of multidirectionally unstable supracondylar humerus fractures in children can shorten the operative time. Another important advantage of this method is the reduced intraoperative fluoroscopy times. Wei et al. [15] found that the number of intraoperative fluoroscopies in the assisted treatment of multidirectionally unstable supracondylar fractures of the humerus in children with olecranon Kirschner wire threading technique was significantly less than that in patients treated with conventional closed reduction. They speculated that the use of joystick technology to assist the reduction of multidirectionally unstable supracondylar humerus fractures in children is a good choice and can reduce the time of fluoroscopy and achieve good reduction effect. However, the disadvantage is that during the operation, the surgeon and his assistant should maintain the reduction state and continue to be exposed to radiation. In this study, the fluoroscopy times were 29.4 ± 9.2 times in the control group and 15.2 ± 6.3 times in the study group. It can be seen that our method can significantly reduce the intraoperative fluoroscopy times; moreover, there is no need to use additional reduction tools or increase X-ray exposure to the surgeon. This method also increases the success rate of closed reduction. It has been reported in the literature that forced and repeated reduction is not recommended for children with multiple intraoperative closed reduction failures, which may lead to iatrogenic soft tissue injury [16], and open reduction should be replaced with the closed reduction. Some scholars believe that there is no significant difference between closed reduction and open reduction internal fixation in fracture stability and functional recovery [17]. However, more scholars believe that closed reduction can achieve better efficacy and lower the occurrence of complications than open reduction [18]. Therefore, to reduce the open reduction rate of fracture, scholars have been exploring better closed reduction techniques. Silva et al. [10] found in the treatment of multidirectionally unstable supracondylar fractures of the humerus in children (type IV) that closed reduction failed in two patients during the operation and open reduction was required, with an open reduction rate of 17% (2/12). In this study, four cases in the control group failed closed reduction and were required to undergo open reduction, with an open reduction rate of 17% (4/23). In the study group, closed reduction was successful, and the open reduction rate was 0%. The results of this study were similar to the surgical reduction effects of Silva et al. [10] and Wei et al. [15].

In this study, the postoperative follow-up results showed that both surgical methods could achieve good appearance and function. There were no significant differences in fracture healing time, BA, SCA, and ROM between the two groups. According to the Flynn elbow function score, 92% of the study group and 86.9% of the control group achieved excellent treatment results. 3 months after surgery, five patients had varying degrees of elbow flexion and extension limitation, and all patients returned to the normal range of activity at the last follow-up after being instructed to strengthen active functional exercise. This is consistent with the findings of Bernthal [19] and Spencer [20] et al., who suggested that functional impairment after elbow fracture can be gradually restored to normal within 1 year after injury. Previous studies have shown that the total incidence of postoperative complications of supracondylar fracture of humerus is about 1%, including vascular and nerve injury, needle tract infection, and angulation deformity [21]. None of the patients in this study experienced surgery-related complications during follow-up, which may be related to the relatively small sample size.

In conclusion, children with multidirectionally unstable supracondylar humerus fractures, Kirschner wire reconstruction with internal and lateral column periosteal hinge-assisted closed reduction and Kirschner wire internal fixation have similar therapeutic effects as closed reduction and Kirschner wire internal fixation, but can shorten the operation time, reduce intraoperative fluoroscopy times, and reduce the incision rate. The strength of this study is that it describes a new surgical technique that simplifies this complex fracture reduction technique.

## Data Availability

All of the material is owned by the authors, and/or no permissions are required.

## References

[1] Dineen HA, Stone J, Ostrum RF (2019). Closed reduction percutaneous pinning of a pediatric supracondylar distal humerus fracture. J Orthop Trauma.

[2] Carter CT, Bertrand SL, Cearley DM (2013). Management of pediatric type III supracondylar humerus fractures in the United States: results of a national survey of pediatric orthopaedic surgeons. J Pediatr Orthop.

[3] Kropelnicki A, Ali AM, Popat R (2019). Paediatric supracondylar humerus fractures. Br J of Hosp Med.

[4] Duffy S, Flannery O, Gelfer Y (2021). Overview of the contemporary management of supracondylar humeral fractures in children. Eur J Orthop Surg Traumatol.

[5] Gartland JJ (1959). Management of supracondylar fractures of the humerus in children. Surg Gynecol Obstet.

[6] Leitch KK, Kay RM, Femino JD (2006). Treatment of multidirectionally unstable supracondylar humeral fractures in children. A modified Gartland type-IV fracture. J Bone Joint Surg Am.

[7] Mitchell SL, Sullivan BT, Ho CA (2019). Pediatric Gartland type-IV supracondylar humeral fractures have substantial overlap with flexion-type fractures. J Bone Joint Surg Am.

[8] Flynn K, Shah AS, Brusalis CM (2017). Flexion-type supracondylar humeral fractures: ulnar nerve injury increases risk of open reduction. J Bone Joint Surg Am.

[9] Iobst CA, Spurdle C, King WF (2007). Percutaneous pinning of pediatric supracondylar humerus fractures with the semisterile technique: the Miami experience. J Pediatr Orthop.

[10] Silva M, Cooper SD, Cha A (2015). The outcome of surgical treatment of multidirectionally unstable (Type IV) pediatric supracondylar humerus fractures. J pediatr orthop.

[11] Flynn JC, Matthews JG, Benoit RL (1974). Blind pinning of displaced supracondylar fractures of the humerus in children Sixteen years’ experience with long-term follow-up. J Bone Joint Surg Am.

[12] Segal D, Cobb L, Little KJ (2020). Fracture obliquity is a predictor for loss of reduction in supracondylar humeral fractures in older children. J Pediatr Orthop B.

[13] Green BM, Stone JD, Bruce RW, Fletcher ND (2017). The use of a transolecranon pin in the treatment of pediatric flexion-type supracondylar humerus fractures. J Pediatr Orthop.

[14] LiBrizzi CL, Klyce W, Ibaseta A (2020). Sex-based differences in pediatric supracondylar humerus fractures. Medicine (Baltimore).

[15] Wei YS, Liu WL, Bai R (2020). The use of a transolecranon pin joystick technique in the treatment of multidirectionally unstable supracondylar humeral fractures in children. J Pediatr Orthop B.

[16] Georgescu I, Gavriliu S, Pârvan A (2013). Burnei’s “double X” internal fixation technique for supracondylar humerus fractures in children: indications, technique, advantages and alternative interventions: Study and Research Group in Pediatric Orthopaedics-2012. J Med Life.

[17] Lee HY, Kim SJ (2007). Treatment of displaced supracondylar fractures of the humerus in children by a pin leverage technique. J Bone Joint Surg Br.

[18] Aktekin CN, Toprak A, Ozturk AM (2008). Open reduction via posterior triceps sparing approach in comparison with closed treatment of posteromedial displaced Gartland type III supracondylar humerus fractures. J Pediatr Orthop B.

[19] Bernthal NM, Hoshino CM, Dichter D, Wong M, Silva M (2011). Recovery of elbow motion following pediatric lateral condylar fractures of the humerus. J Bone Joint Surg Am.

[20] Spencer HT, Wong M, Fong YJ, Penman A, Silva M. Prospective longitudinal evaluation of elbow motion following pediatric supracondylar humeral fractures. J Bone Joint Surg Am. 2010;92:904–10. 10.2106/JBJS.I.00736 20360514

[21] Zorrilla S, de Neira J, Prada-Cañizares A, Marti-Ciruelos R, Pretell-Mazzini J (2015). Supracondylar humeral fractures in children: current concepts for management and prognosis. Int Orthop.

